# Brain Network Organization in Focal Epilepsy: A Systematic Review and Meta-Analysis

**DOI:** 10.1371/journal.pone.0114606

**Published:** 2014-12-10

**Authors:** Eric van Diessen, Willemiek J. E. M. Zweiphenning, Floor E. Jansen, Cornelis J. Stam, Kees P. J. Braun, Willem M. Otte

**Affiliations:** 1 Department of Pediatric Neurology, Brain Center Rudolf Magnus, University Medical Center Utrecht, Utrecht, The Netherlands; 2 Department of Clinical Neurophysiology, VU University Medical Center, Amsterdam, The Netherlands; 3 Biomedical MR Imaging and Spectroscopy Group, Image Sciences Institute, University Medical Center Utrecht, Utrecht, The Netherlands; Hospital for Sick Children, Canada

## Abstract

Normal brain functioning is presumed to depend upon interacting regions within large-scale neuronal networks. Increasing evidence exists that interictal network alterations in focal epilepsy are associated with cognitive and behavioral deficits. Nevertheless, the reported network alterations are inconclusive and prone to low statistical power due to small sample sizes as well as modest effect sizes. We therefore systematically reviewed the existing literature and conducted a meta-analysis to characterize the changes in whole-brain interictal focal epilepsy networks at sufficient power levels. We focused on the two most commonly used metrics in whole-brain networks: average path length and average clustering coefficient. Twelve studies were included that reported whole-brain network average path length and average clustering coefficient characteristics in patients and controls. The overall group difference, quantified as the standardized mean average path length difference between epilepsy and control groups, corresponded to a significantly increased average path length of 0.29 (95% confidence interval (CI): 0.12 to 0.45, p = 0.0007) in the epilepsy group. This suggests a less integrated interictal whole-brain network. Similarly, a significantly increased standardized mean average clustering coefficient of 0.35 (CI: 0.05 to 0.65, p = 0.02) was found in the epilepsy group in comparison with controls, pointing towards a more segregated interictal network. Sub-analyses revealed similar results for functional and structural networks in terms of effect size and directionality for both metrics. In addition, we found individual network studies to be prone to low power due to the relatively small group differences in average path length and average clustering coefficient in combination with small sample sizes. The pooled network characteristics support the hypothesis that focal epilepsy has widespread detrimental effects, that is, reduced integration and increased segregation, on whole brain interictal network organization, which may relate to the co-morbid cognitive and behavioral impairments often reported in patients with focal epilepsy.

## Introduction

Traditionally, the brain has been perceived as a set of brain areas with highly specialized functions. However, there is increasing evidence that brain functioning is emerging from a complex interplay of different brain areas. This shift from a ‘location specific’ to a more ‘network oriented’ approach has revealed novel insights into the physiological functioning of the brain and further clarified neurological diseases, including epilepsy [1], [2]. Several studies have shown a relation between epilepsy and disrupted functional and structural brain networks. This relation could, at least partly, be attributed to the cognitive and behavioral impairments often found in patients with epilepsy [3], [4], [5], [6].

**Figure 1:**
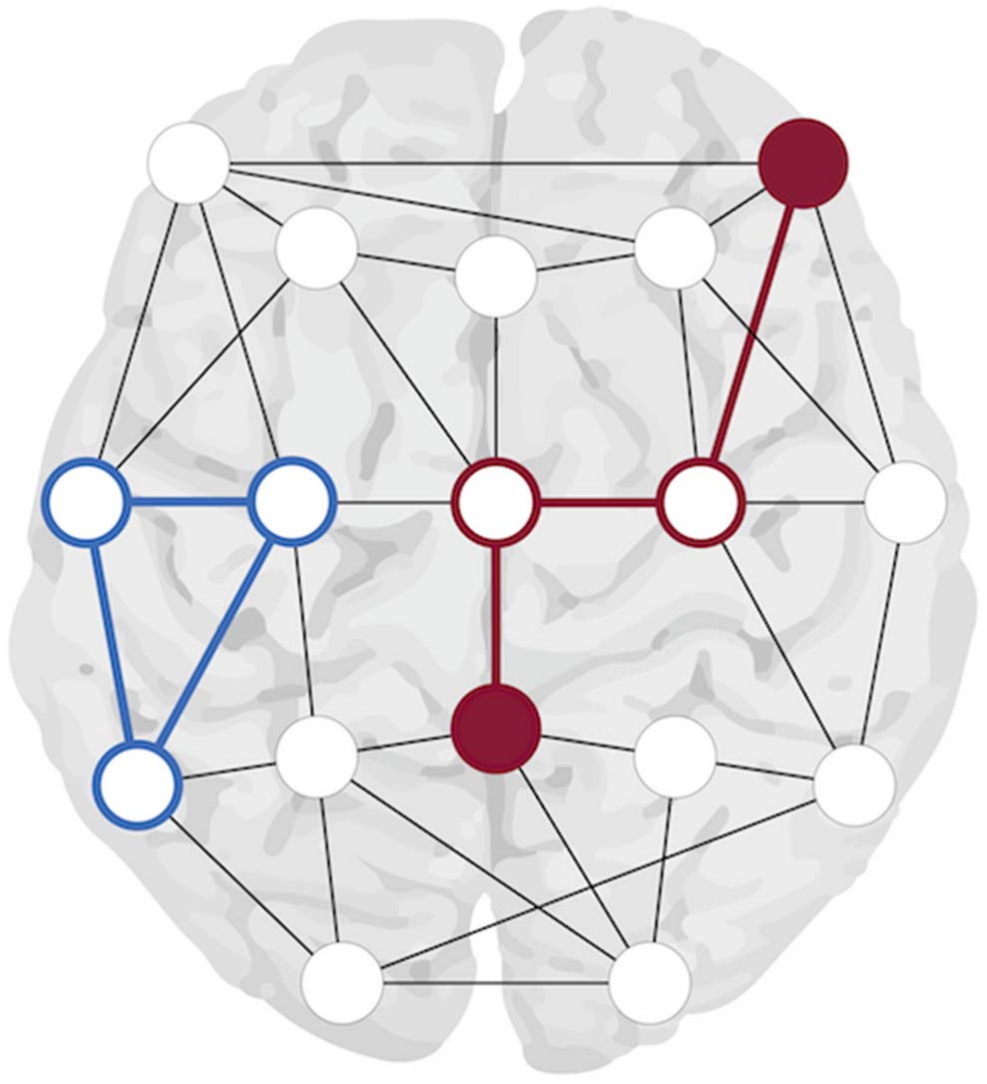
Clustering coefficient and path length. Explanation of the clustering coefficient and path length using a schematic whole-brain binary network representation of nodes (circles) and binary edges (black lines). The clustering coefficient is based on triangles (one triangle shown in blue) and is equivalent to the fraction of the node’s neighbors that are also neighbors of each other. The average clustering coefficient is a global measure of network segregation and reflects the clustered connections around individual nodes. The path length in a binary network is the minimal number of edges that must be traversed to travel from one node to another. In red an example path is given which contains the minimal number of edges (i.e. three) between the two uniform red nodes. The average path length, a global measure of network integration, is this average minimal number of edges of all possible node connections.

To quantify and characterize brain networks, studies are increasingly using network analysis as a mathematical paradigm [7], [8], [9], [10]. Network analysis reduces complex systems to a collection of ‘nodes’ (that is, brain areas) and ‘edges’ (that is, connections between brain areas). From these elementary network building blocks various quantitative metrics can be inferred. Two very informative metrics, the path length and clustering coefficient, have been widely used to characterize brain network organization and changes herein in healthy and epileptic brains (Fig. 1). Path length refers to the minimal number of edges that must be traversed to travel from one node in the network to another. The average path length of a network is inversely related to the level of network integration. The clustering coefficient is defined as the connection probability of nearest neighbor nodes and the average clustering coefficient represents network segregation. A highly segregated network has a high average clustering coefficient. A short average path length and a high average clustering coefficient characterize healthy brain networks: a so-called small-world configuration [8], [10]. A small-world configuration is considered optimal for network functioning as the number of long distance connections is minimized while high average clustering of neighboring nodes is retained. This reduces the network’s ‘building and maintenance costs’ without compromising fast exchange of information [2].

It is important to accurately quantify changes in brain network organization to increase our understanding of epilepsy and its associated cognitive and behavioral comorbidities. Both clinical and experimental neurophysiological and imaging studies have, in general, reported a less optimal interictal network organization in patients with epilepsy. Nevertheless, these case-control studies have reported contradictive results. Increased, decreased or unchanged average path length and/or average clustering coefficient have been described in patients with epilepsy as compared to controls; for overviews see [4], [11], [12]. A possible explanation for the inconclusive network characterization, as found in the individual network studies comparing focal epilepsy with controls, is a lack of sufficient statistical power. The number of included subjects is typically in the range of 20–40 per group. As a consequence the chance of detecting a true effect might be reduced and, consequently, results less reliable [13].

In this meta-analysis we first aimed to obtain an accurate estimate of the changes in interictal network organization in patients with epilepsy as compared to healthy individuals using existing literature. The advantage of a meta-analysis over a qualitative review is that it enables a quantitative analysis of the current literature and provides data on the between-study heterogeneity. We were exclusively interested in network studies including patients with focal epilepsy as these often have cognitive and behavioral impairments that are not necessarily directly associated with the epileptogenic focus [14], [15]. This implicates that brain areas outside the epileptogenic focus are affected as well, which is supported by the widespread changes of white matter structure that have been found in focal epilepsy using diffusion tensor imaging [16]. Network analysis offers a unique perspective to investigate the complex involvement of the different brain areas in focal epilepsy outside the epileptogenic zone. Second, to help to increase reproducibility of – and reduce spurious findings in – future focal epilepsy network studies, we aimed to estimate the statistical power of existing literature and proposed the minimal sample size required to have sufficiently powered future network studies comparing focal epilepsy with healthy individuals.

## Methods

### Information sources and search strategy

Studies were identified by searching the online databases Pubmed (NCBI), ISI Web of Science (Thomson Reuters) and Embase (Excerpta Medica Database), and reviewing the reference lists of selected studies. Language was restricted to English. Search terms related to epilepsy, different neurophysiological and imaging studies, and network analysis were used. Details on the search strategies are presented in S1 Table in File S1. The search was conducted January 16^th^, 2014.

### Selection criteria

We included studies that compared brain networks of focal epilepsy patients with controls using the average path length, average clustering coefficient or both, in networks not restricted to a specific part of the brain (whole-brain networks), irrespective of acquisition technique.

We allowed included studies to differ in various ways, such as in acquisition technique and network construction. Multiple clinical neurophysiology and imaging techniques are available to infer functional or structural connectivity between brain regions and construct networks. Functional connectivity may be derived from synchronized neuronal activity recorded from distinct brain regions using electroencephalography (EEG), magnetoencephalography (MEG) or resting-state functional MRI. Diffusion tensor imaging is an MRI technique that enables the characterization of directional water diffusion in white matter bundles. It might be exploited to map structural connectivity between remote brain regions using tractography [17]. Another means to infer structural connectivity from MRI data is to correlate cortical thicknesses between functional fields [18].

When network analysis was introduced in the field of neuroscience, most studies relied on methods that calculated the average path length and average clustering coefficient without taking the strength (or ‘weight’) of network connections into account. This is called a binary analysis. The construction of binary networks depends on an arbitrary threshold. A high threshold results in a network with a high level of sparsity (that is, only the strongest connections are retained), and vise versa. More recently threshold-free methods are favored that use the connection strength to calculate these two network metrics, which is called weighted analysis [7]. In addition to these methodological differences a subset of studies normalized their network metrics for statistical comparison, using null models [19]. This normalization might enhance statistical comparability between groups within studies [20]. In our statistical analysis we took these differences between studies as much as possible into account using *i*) a standardized mean difference measure, *ii*) a random-effect model, *iii*) sensitivity analyses and *iv*) a leave-one-out validation method (the analysis is explained below).

Study exclusion criteria were: network analysis of a specific part of the brain only (that is, no whole brain networks; this includes corticography studies), no data on average path length and average clustering coefficient, no comparisons with controls, brain surgery in the focal epilepsy group, or different studies using an identical patient population.

### Data extraction

Two authors independently performed the literature search and screened all study titles and abstracts. From the eligible articles, full-text versions were retrieved and reviewed. Data were extracted by one author [WZ] and checked by another [EvD]. For missing data the corresponding authors were contacted. Disagreements on study inclusion and data extraction were resolved by reaching consensus through discussion with a third author [WMO]. The following data was extracted:

#### Study population

Group sizes, sex distribution, age at data acquisition, age at epilepsy onset, duration of epilepsy, antiepileptic drug usage and presumed etiology.

#### Acquisition

Technique specific information such as modality, field strength (for imaging studies) and sampling frequency (for neurophysiological studies).

#### Network construction

Network size, connectivity measure, binary or weighted connections and sparsity level if binary connections are used.

#### Network metrics

The mean and standard deviations (SD) of the average path length and average clustering coefficient are used. If SDs were not provided we calculated those from the standard error or 95% confidence intervals (CIs). If a range of sparsity levels was given, values corresponding to the lowest, middle and highest level were extracted. For studies reporting on both binary and weighted networks, all data were extracted (we used the binary data for the sensitivity analysis and included the weighted data for the summary estimates). Normalized and non-normalized measures were extracted if both were reported. If network metrics were reported for subgroups only, they were combined into a single value using weighted mean calculation. The average path length was defined as the reciprocal of the network efficiency if studies reported the latter only [21]. We did not differentiate between slightly different mathematical formulations of the average clustering coefficient that exist in literature (for instance, the global [22] or the averaged local [23] clustering coefficient).

EEG and MEG network studies often report network metrics for different frequency bands. For those studies the average path length and average clustering coefficient values were only extracted for the theta frequency band networks, as several studies have shown that network alterations in patients with focal epilepsy are most prominent in the theta frequency band [24], [25], [26], [27], [28], [29], [30].

### Quality assessment

Study quality was assessed independently by two authors [WZ, WMO] with the adjusted Newcastle-Ottawa scale method [31]. Disagreements were resolved by consensus. This scale is developed for non-randomized studies and allocates stars to the following domains: the selection and comparability of study groups, and the ascertainment for the exposure of interest for case-control or the outcome of interest for cohort studies. A star system is used in the Newcastle-Ottawa scale for semi-quantitative analysis wherein high quality studies get more stars, with a maximum of seven in total.

### Methods of analysis

All data were entered in RevMan version 5.2.6 for analysis (http://ims.cochrane.org/revman). Average path length and average clustering coefficient summary group differences, and their CIs, were calculated by fitting random-effect weighted standardized mean difference models using restricted maximum likelihood estimation [32]. A random-effect model was used because this provides a more conservative effect than a fixed-effect model as it takes heterogeneity between studies into account. We used the standardized mean difference because we expected that scale differences of the average path length and average clustering coefficient values between studies might be affected by modality, connectivity measure, network construction (for example, binary or weighted, normalization or no normalization) and sparsity level. The standardized mean difference model is a well-excepted method to calculate summary values if scale variation is present. It has the advantage of resulting in a scale-free summary estimate (that is, free of the original measurement scale) [33]. Significant heterogeneity between studies was defined by *p* value <0.1 using the χ^2^ test or an I^2^>75%. An I^2^ value below 40% suggests negligible heterogeneity, between 40 and 75% moderate heterogeneity, and above 75% considerable heterogeneity [34]. In the latter case, summary estimates are unreliable. To detect a reporting bias in the included studies, we visually inspected funnel plots for asymmetry and excluded outliers [35]. Furthermore, we checked whether individual studies had a significant effect on the heterogeneity by means of a leave-one-out analysis (that is, omitting one study at the time with recalculation). We excluded studies with significant effects on summary estimations. Furthermore, we performed meta-regression to explain the potential heterogeneity in the standardized mean differences of the average clustering coefficient and average path length. We regressed the individual study standardized mean difference values against the *i*) mean age of the patients, control subjects and ages from patients and controls combined, and the *ii*) mean duration of epilepsy.

### Subgroup and sensitivity analyses

We distinguished overall changes in functional and structural networks using sub-analyses based on modality: functional synchronization for functional networks and white matter or cortical thickness connectivity for structural networks. Considering the brain network alterations during lifespan [36], [37], we performed separate sub-analyses comparing children with adults. To investigate the influence of different types of epilepsy on network alterations, we conducted separate analyses for temporal lobe epilepsy, extratemporal epilepsy and patients with epilepsy secondary to brain tumors. The robustness of the summary estimates was tested by means of sensitivity analysis. In this analysis, changes in point estimates and CIs were determined using recalculations at different network sparsity levels for binary or weighted networks.

### Statistical power

We calculated the power of each individual study using the estimated summary effect obtained from the random-effect analysis to which it contributed. Power calculations were done with the *pwr* package (http://cran.r-project.org/web/packages/pwr) of the R software. The individual study powers for the average path length and average clustering coefficient are presented as mean ± SD.

## Results

### Article selection

**Figure 2:**
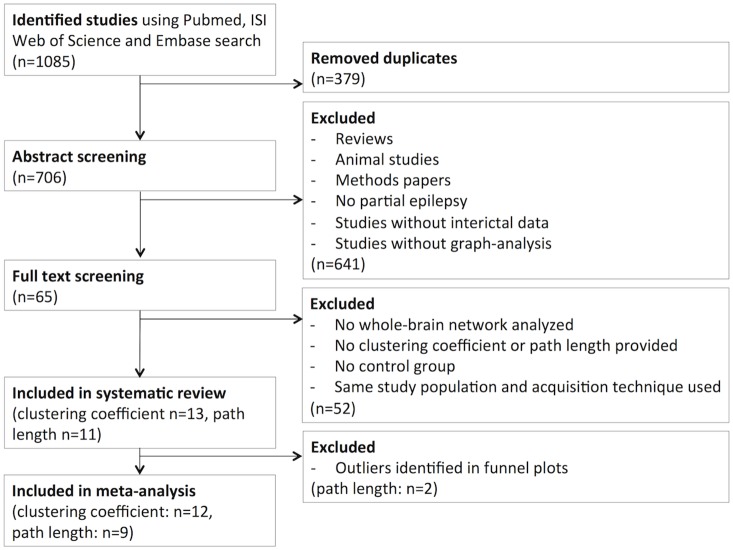
Flow chart. Flow chart of the literature search and identification of studies for inclusion in the meta-analysis.

The literature search and study selection flow chart is given in Fig. 2. We found no previous systematic literature reviews or meta-analyses on network organization in focal epilepsy. From 65 manuscripts, full text was reviewed and reference lists were inspected. Eventually, thirteen studies were initially included [21], [25], [29], [30], [38], [39], [40], [41], [42], [43], [44], [45], [46]. Study characteristics are summarized in Table 1; more detailed methodological information on the included electrophysiological and functional MRI studies is provided in S2 Table in S1 File. Two of the included studies only reported on the average clustering coefficient and not the average path length [40], [45].

**Table 1:** Sample characteristics and network properties of the studies included in the systematic review.

Study	Characteristics of study population							Imaging characteristics		Network properties				
	Group	Groupsize (N)	Female/Male	Age inyears(±SD)	Durationin years(±SD)	Type oflesion	Drugtreatment	Modality	Field strengthor samplefrequency	Network size (template)	Connectivitymeasure	Binary/ weighted	Sparsitylevel	Used networkcharacteristics
Bartolomei et al., 2006	Tumor	17	9/8	40 (12)	n.r.	Mixed	n.r.	MEG	312.5 Hz	149	SL	Binary	k = 10	γ, λ
	Control	15	7/8	31 (8)										
Bernhardtet al., 2011	TLE	122	70/52	36 (10.5)	20.5 (12)	Hippocampalatrophy	n.r.	MRI	1.5 Tesla	52 (AAL)	Cortical thickness correlations	Binary	5–40%	C, L, γ, λ, σ, BC
	Control	47	24/23	32 (12)										
Bonilhaet al.,2012	TLE	12	8/4	37.5 (9.8)	n.r.	Hippocampalatrophy	n.r.	DTI	3.0 Tesla	20 (AAL)	Fiberdensity	Binary	5–55%	C, Eglobal,Elocal, BC
	Control	26	16/20	34.3 (8.8)										
Bosmaet al.,2009	Tumor	17	n.r.	42.7 (11.2)	n.r.	LGG	Mono- orpolytherapy	MEG	312.5 Hz	149	PLI	Binary	k = 10	C, L, γ, λ, σ, K
	Control	17	n.r.	42.6 (12.7)										
Horstmannet al.,2010	Symptomaticfocal epilepsy	21	12/9	38 (11)	21.3 (11)	Mixed	Mono- orpolytherapy	EEG	254.31 Hz	29	Time-resolvedphasesynchronization	Binary &weighted	k = 3–7;lowestconnectedness	C, L, R,
	Control	23	11/12	33 (9)										
Liao et al.,2010	TLE	18	7/11	23.9 (8.5)	13.2 (10)	Hippocampalsclerosis	Mono- orpolytherapy	fMRI	1.5 Tesla	90 (AAL)	Pearson’scorrelationcoefficient	Binary	0.22–0.386	C, L, γ, λ, σ, K
	Control	27	8/19	25.6										
Quraan et al.,2013	TLE	9	2/7	42 (13)	n.r.	Mixed	Polytherapy	EEG	500 Hz	64	Meanphasecoherency	Binary	0.3≤T≤0.6; 18.9≤K≤37.8	γ, Eglobal, σ
	Control	15	n.r.	33 (10)										
Raj et al.,2010	TLE	27	17/10	38.5 (7.7)	n.r.	Mixed	Mono- orpolytherapy	MRI	4.0 Tesla	64	Cortical thickness correlations	Binary	15%	C, K, Entropy
	Control	30	20/10	38.3 (9.7)										
Vaessenet al.,2012	Focal epilepsy	39	30/19	40 (12)	17.8 (11.1)	Mixed	Mono- orpolytherapy	DTI	3.0 Tesla	90 (AAL)	Structuralconnectionstrength	Weighted	Voxelstransversedby>50 tracts	C, L
	Control	23	14/9	40 (13)										
Vaessen et al.,2013	FLE	28	n.r.	11.3 (1.5)	n.r.	Mixed	n.r.	fMRI	3.0 Tesla	82	Pearson’scorrelationcoefficient	Binary &weighted	0.35–0.75	C, L
	Control	37	n.r.	10.5 (1.3)										
Van Dellenet al.,2012	Tumor	35	15/20	42.2 (9.5)	7.4 (6.5)	Mixed	n.r.	MEG	625 Hz	136	PLI	Weighted	Connectionstrength	γ, λ, Q
	Control	36	18/18	43.9 (11.9)										
Van Diessenet al.,2013	Focal epilepsy	35	11/24	10.1 (3.4)	0	Mixed	none	EEG	512 Hz	17	SL	Weighted	Connectionstrength	C, L, BC, K, CC, EC
	Control	35	11/24	9.9 (3.1)										
Vlooswijket al.,2011	Focal epilepsy	41	21/20	40 (12)	17.8 (11)	Mixed	Mono- orpolytherapy	fMRI	3.0 Tesla	893 (AAL-adapted)	Pearson’scorrelationcoefficient	Binary	0.87–1	C, L, γ, λ,Eglobal, Elocal
	Control	23	14/9	40 (12)										

TLE = temporal lobe epilepsy, FLE = frontal lobe epilepsy, n.r. = not reported, LGG = low-grade glioma, AAL = anatomical automatic labeling, SL = synchronization likelihood, PLI = phase lag index, C = average clustering coefficient, L = average path length, γ = normalized clustering coefficient, λ = normalized average path length, Q = modularity, BC = betweenness centrality, K = degree, CC = closeness centrality, E_global_ = global efficiency, E_local_ = local efficiency.

The quality of included studies was variable with a range in Newcastle-Ottawa scores between five and seven stars (see S3 Table in S1 File). Based on the asymmetry in the average path length funnel plot, as shown in = S1 Figure in S1 File, we excluded two obvious outliers from the average path length data from the final analysis [29], [46]. No obvious outliers were detected in the average clustering coefficient funnel plot, shown in S2 Figure in S1 File. We tested the remaining studies on their individual contribution to heterogeneity.

All data are provided in the Supporting Information (S1 Data).

### Average path length

**Figure 3:**
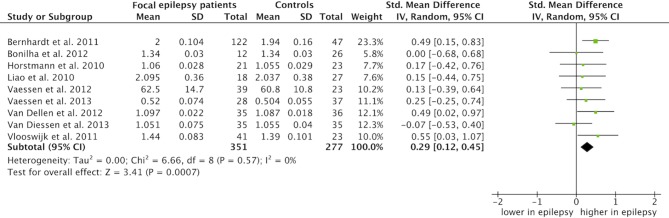
Meta-analysis of the average path length. The forest plot displays the standardized mean differences of the average path length between focal epilepsy patients and controls with the 95% confidence intervals (CI). No difference is specified with a vertical line at 0. The overall pooled SMD was 0.29 (CI: 0.12 to 0.45, p = 0.0007), that is, a significant higher average path length was observed in whole brain networks of focal epilepsy patients relative to controls.

Average path length summary estimates were based on data from nine studies [21], [25], [30], [38], [39], [41], [42], [43], [44]. The standardized mean difference summary estimate, based on 351 patients with focal epilepsy and 277 control networks was 0.29 (CI: 0.12 to 0.45, p = 0.0007; Fig. 3). In other words, the network organization in patients with focal epilepsy was characterized by an increased average path length as compared to controls. There was no evidence for heterogeneity: I^2^ = 0%, p = 0.57 (Table 2). Data for the summary estimate was taken from normalized weighted network analysis, or the highest sparsity level if the study used binary networks only. Sensitivity analysis revealed only very small differences (that is, in the order of 1–10%) in summary estimates and CIs and *p* values if we changed the type of network or sparsity level (see S4 Table in S1 File).

**Table 2:** Leave-one-out analysis.

	Average path length			Average clustering coefficient		
Excluded study	SMD (CI)	I^2^ (%)	?^2^ (p-value)	SMD (CI)	I^2^ (%)	?^2^ (p-value)
***None***	***0.29 (0.12, 0.45)***	***0***	***0.57***	***0.35 (0.05, 0.65)***	***73***	***<0.0001***
Bernhardt et al., 2011	0.22 (0.04, 0.41)	0	0.68	0.32 (−0.01, 0.66)	73	<0.0001
Bonilha et al., 2012	0.30 (0.13, 0.47)	0	0.55	0.29 (−0.01, 0.59)	71	0.0001
Bosma et al., 2009				0.31 (−0.00, 0.62)	74	<0.0001
Horstmann et al., 2010	0.30 (0.12, 0.47)	0	0.48	0.36 (0.04, 0.69)	75	<0.0001
Liao et al., 2010	0.30 (0.13, 0.47)	0	0.49	0.36 (0.03, 0.69)	75	<0.0001
Quraan et al., 2013				0.37 (0.05, 0.69)	75	<0.0001
Raj et al., 2010				0.29 (−0.01, 0.60)	71	0.0001
Vaessen et al., 2012	0.30 (0.13, 0.48)	0	0.51	0.40 (0.09, 0.72)	73	<0.0001
Vaessen et al., 2013	0.29 (0.12, 0.46)	0	0.47	0.37 (0.04, 0.70)	75	<0.0001
van Dellen et al., 2012	0.26 (0.08 0.43)	0	0.56	0.31 (−0.01, 0.63)	73	<0.0001
van Diessen et al., 2013	0.33 (0.16, 0.51)	0	0.76	0.40 (0.08, 0.72)	73	<0.0001
Vlooswijk et al., 2011	0.26 (0.08, 0.43)	0	0.59	0.44 (0.18, 0.71)	61	0.004

The leave-one-out analysis results are shown in this table. The effect of leaving out one study on the overall heterogeneity is presented as the recalculated standardized mean differences (SMD) (after removal of the study in the first column) and the 95% confidence interval (CI) and corresponding I^2^ statistic and χ^2^ test *p*-value.

### Average clustering coefficient

**Figure 4:**
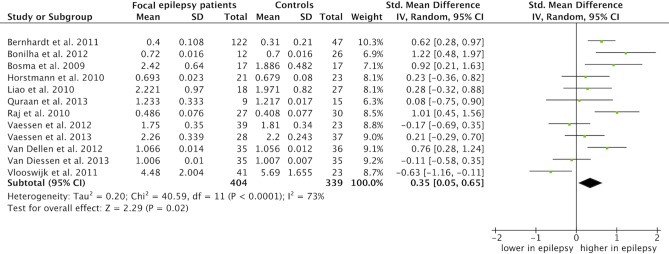
Meta-analysis of the average clustering coefficient. The forest plot displays the standardized mean differences (SMD) of the average clustering coefficient between focal epilepsy patients and controls with the 95% confidence intervals (CI). No difference is specified with a vertical line at 0. The overall pooled SMD was 0.35 (CI: 0.05 to 0.65, p = 0.02), that is, a significant higher average clustering coefficient was observed in whole brain networks of focal epilepsy patients relative to controls.

Average clustering coefficient summary estimates were based on twelve studies [21], [25], [30], [38], [39], [40], [41], [42], [43], [44], [45], [46]. The standardized mean difference summary estimate, including 404 networks from focal epilepsy patients and 339 control networks, was 0.35 (CI: 0.05 to 0.65, p = 0.02; Fig. 4). Stated differently, the network organization in patients with focal epilepsy was characterized by an increased average clustering coefficient as compared to controls. There was moderate heterogeneity in the data: I^2^ = 73%, p<0.0001 (Table 2), indicating that the summary estimate might be unreliable. Results were, similar to the average path length estimate, stable in the sensitivity analysis (see S4 Table in S1 File).

### Meta-regression average path length and average clustering coefficient

No significant relation was found between the standardized mean difference values (for average path length and average clustering coefficient) and the mean age (patients, controls or both) or mean duration of epilepsy.

### Subgroup analyses

**Figure 5:**
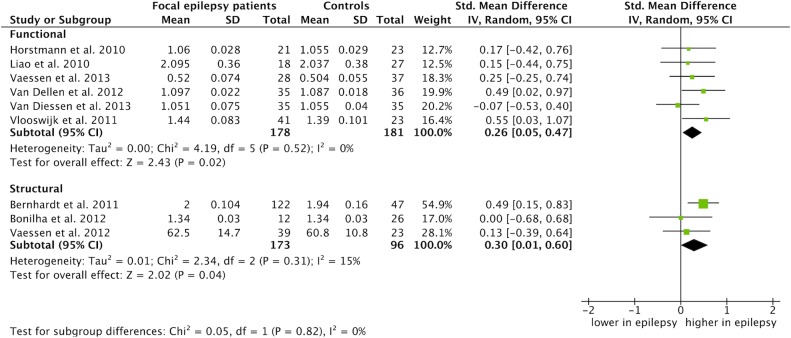
Meta-analysis of the average path length separated by network modality. The forest plot displays the standardized mean differences (SMD) between focal epilepsy patients and controls with the 95% confidence intervals (CI) for the functional and structural network studies reporting the average path length. No difference between patients and controls is specified with a vertical line at 0. The overall pooled SMD for the functional average path length was 0.26 (CI: 0.05 to 0.47, p = 0.02) and 0.30 for the structural average path length (CI: 0.01 to 0.60, p = 0.04). The SMDs of these subgroups were not statistically different (p = 0.82, I^2^ = 0%).

**Figure 6:**
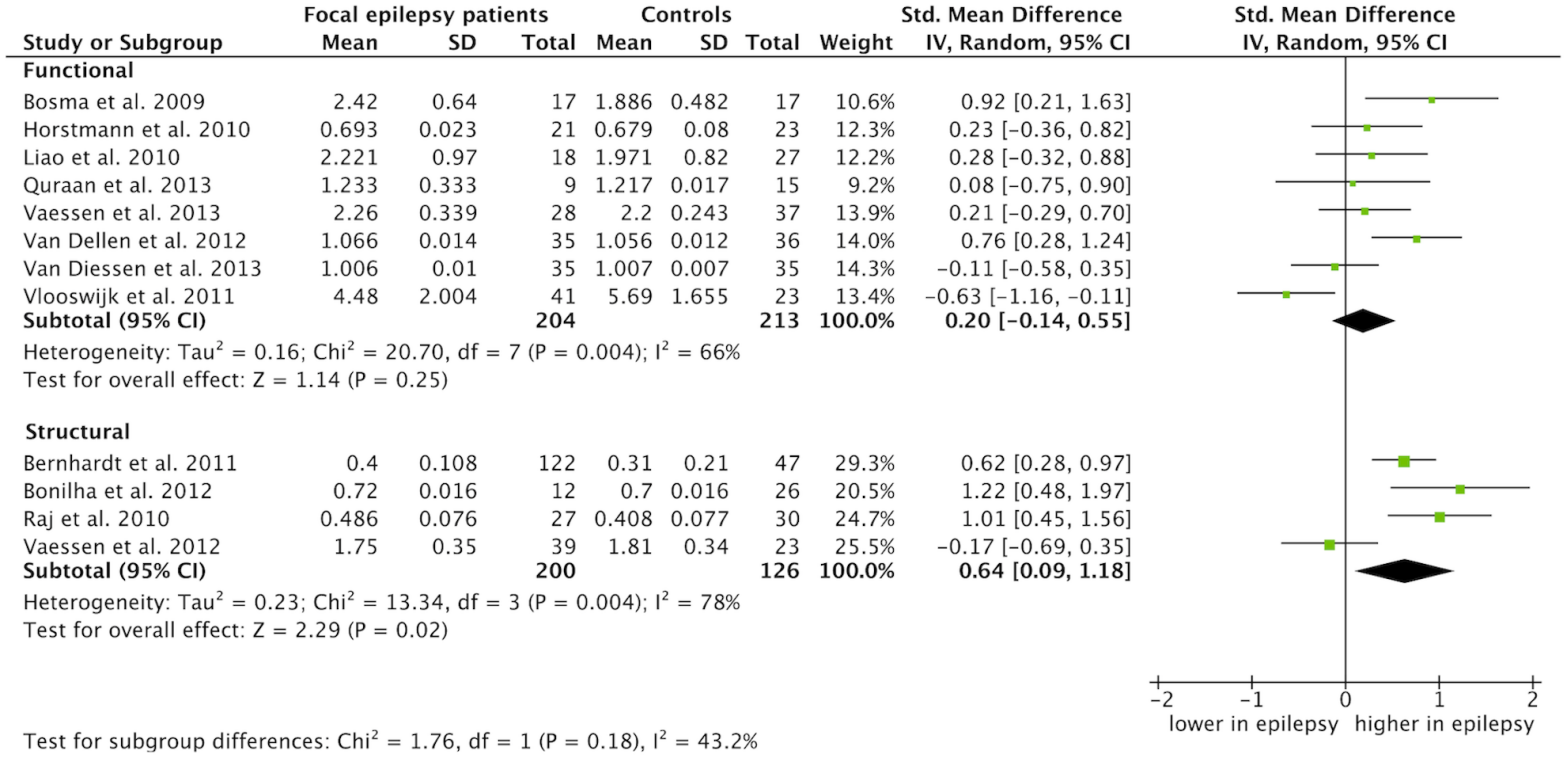
Meta-analysis of the average clustering coefficient separated by network modality. The forest plot displays the standardized mean differences (SMD) between focal epilepsy patients and controls with the 95% confidence intervals (CI) for the functional and structural network studies reporting the average clustering coefficient. No difference between patients and controls is specified with a vertical line at 0. The overall pooled SMD for the functional average clustering coefficient was 0.20 (CI: −0.14, 0.55, p = 0.25) and 0.64 for the structural average clustering coefficient (CI: 0.09 to 1.18, p = 0.02). The SMDs of these subgroups were not statistically different (p = 0.18, I^2^ = 43.2%).

Modality specific summary estimates for the average path length and average clustering coefficient are provided in Fig. 5 and 6, respectively. Both functional and structural focal epilepsy networks were characterized by a significantly increased average path length as compared to control networks: 0.26 for the functional (CI: 0.05 to 0.47, p = 0.02) and 0.30 for the structural networks (CI: 0.01 to 0.60, p = 0.04) (Fig. 5). The average clustering coefficient was significantly increased in structural focal epilepsy networks: 0.64 (CI: 0.09 to 1.18, p = 0.02). There was no significant difference in average clustering coefficient for the functional epilepsy networks: 0.20 (CI: −0.14, 0.55, p = 0.25) (Fig. 6). Age group specific estimates revealed an increased average path length and average clustering coefficient only for the adults with epilepsy in comparison with controls (see S3a Figure and S3b Figure in S1 File). No difference in average path length (p = 0.18) and average clustering coefficient (p = 0.13) was found between adults and children with epilepsy. Specific estimates for the subgroup analysis for different types of epilepsy revealed an increased average path length and clustering coefficient for the temporal lobe epilepsy patients only in comparison with controls. There was no difference between the temporal lobe, extratemporal and brain tumor groups in terms of average path length (p = 0.48). The standardized mean differences were significantly different for the average clustering coefficient (p = 0.0002) with an evidently increased segregation for the temporal lobe epilepsy subgroup, but due to a considerable heterogeneity (I^2^ = 88.1%), not reliable (see S4a Figure and S4b Figure in S1 File).

### Statistical power

The average statistical power of included studies was (very) low: 20.8±7.6 and 25.5±10.3% for the average path length and average clustering coefficient, respectively (note that the standard recommended statistical power is in the range of 80 to 90%). Based on the average path length standardized mean difference of 0.29, at least 188 subjects per group are required to obtain a power of 80%. A group size of 251 is required to reach a power of 90%.

## Discussion

In this systematic review and meta-analysis we obtained information on an issue that has not been unequivocal: the network organization in patients with focal epilepsy as compared to healthy individuals. Previous qualitative reviews have identified that case-control studies investigating interictal networks often reveal contradictive results [4], [11]. Here, we tried to remove this ambiguity by means of a quantitative estimate of the group differences in average path length, a measure of network integration, and average clustering coefficient, a measure of network segregation. Interictal brain networks in patients with focal epilepsy are characterized by a significant increase in both average path length and average clustering coefficient, compared to healthy controls. Finally, the statistical power of the included individual network studies was low.

This systematic review adds unique information to previous qualitative reviews [3], [4], [5], [47]. First, it clarifies the type of network change in focal epilepsy interictally: whole brain networks of patients are characterized by a less integrated and more segregated organization (that is, a more regular network organization) than healthy controls. Interestingly, a shift towards an even more regular organization is found during the ictal phase, relative to the interictal phase (for review, see [4], [11]). Studies focusing on dynamic network changes in epilepsy – namely before, during and directly after a seizure – have shown that a shift in network organization towards a more regular state increases further during a seizure and eventually returns to pre-ictal values [48], [49], [50]. Possibly, the regular interictal network organization facilitates fast spreading activity in the brain [48], [50] making it more susceptible to seizures. Second, our results suggest that (frequent) epileptic seizures may have widespread detrimental effects on the structural and functional integrity of interictal brain networks. Data supporting this hypothesis comes from studies characterizing white matter integrity in focal epilepsy, for overview see [51]. A recent systematic review and meta-analysis of clinical studies provided evidence for widespread structural white matter damage distant from the epileptogenic zone [16]. Similarly, an animal study revealed that diffuse, widespread damage to the connecting white matter tracts coincides with reduced functional connectivity and network efficiency [52]. The pathophysiological mechanism of the remote white matter damage remains unknown. Potential explanations include Wallerian degeneration, axonal swelling caused by frequent recurrent seizures, decreased axonal density, increasing permeability of axonal membranes and demyelination [53], [54]. Additionally, it remains unclear, whether epileptic seizures cause the (interictal) network organization to change or if seizures are merely an epiphenomenon of epileptogenic networks [25], [28]. Previous research has revealed that (remote) white matter abnormalities coincide with cognitive impairments in focal epilepsy [55], [56]. More recently, Vaessen and colleagues found distinct structural network changes in cognitively impaired patients with epilepsy, while non-impaired patients did not show network alterations [43]. Interestingly, subgroup analysis in our current study showed that both functional and structural network alterations point in the same direction, although the number of network studies looking at white matter connectivity was too small to draw a firm conclusion. The interplay between structural and functional network alteration is essential to understand the cognitive and behavioral impairments that occur in a considerable part of patients with epilepsy. Future studies, using simultaneous acquisition of functional and structural connectivity, are required to address this issue [57], [58], [59]. We found no difference between adults and children with epilepsy, however the number of included studies is too small to draw a firm conclusion. More studies on children with epilepsy are needed to clarify if, and to what extent, the developing brain organizes itself differently in comparison with the adult brain when epilepsy manifests. Similarly, subgroup analyses for different types of epilepsy did not reveal significant differences between temporal lobe, extratemporal and brain tumor patients, but heterogeneity in the data might obfuscate a true difference. Increasing the number of studies per subgroup could provide better insights to what extent these subgroups have a different network organization. In addition, we performed meta-regression to explain part of the variation in average path length and average clustering coefficient between the individual studies. We expected differences in network organization to relate to subjects’ age and duration of epilepsy (for instance, more damage – and consequently an even less efficient network organization – if a brain is longer exposed to recurrent seizures). However, we found no significant association. Information on other network modifiers such as antiepileptic drug treatment, age at onset, type of epilepsy, presence or absence of a structural lesion, and location of the epileptogenic focus could also be useful in obtaining more details on the relationship between network organization and focal epilepsy, but these patient characteristics were not reported in most of the included studies. Meta-regression was restricted to the mean values reported per study, masking individual subject values. This, or the small number of regression points, could explain the lack of association between patient characteristics and network metrics. A solution to mean regression is individual patient data (IPD) meta-analysis, which is known to provide more reliable estimates than standard meta-analysis, but requires the collection of the original networks and patient datasets for all studies, which is very time consuming and requires active participation of the corresponding authors of all included studies [60].

Our power analysis results suggest that future studies that aim to characterize the network organization in terms of average path length and average clustering coefficient in focal epilepsy should consider minimal sample sizes of 188 per group, as the effect sizes of these two specific network metrics were found to be small. Collaborations between academic institutions could help to obtain the required sample sizes. This strategy has been successful in increasing the reliability of study results in the field of genetic epidemiology [61]. The Human Connectome Project, with over a thousand combined functional MRI scans, is a first successful attempt to boost statistical power in the field of neuroscience and could also serve as an example for future network studies [62]. Other possibilities to decrease the required sample size are to increase the homogeneity of patients, as was shown for the temporal lobe epilepsy subgroup analysis (S4a Figure and S4b Figure in S1 File), or to use more advanced network metrics such as the modularity [25], [44]. Using the minimum spanning tree approach, which focuses on the connectivity backbone of the network, might also help to increase study power as these network backbones are less susceptible to noise [63], [64], [65].

Our study has limitations. Despite the important and intuitively appealing network metrics we used in this study, more information can be obtained from the network organization apart from the average path length and average clustering coefficient. In fact, these two metrics are highly correlated, arguing for more and distinct features in characterizing focal epilepsy networks [66]. Also, average path length and average clustering coefficient provide only information on global network topology. Eligible network features are *i*) the long-tailed degree distribution compatible with the existence of super connected nodes, *ii*) the modular community structure, that is, multiple network groups of densely interconnected nodes, *iii*) usage of the minimum spanning tree approach, a robust method that enables comparison of different types of networks [67] and *iv*) the rich-club ordering, which is the tendency of nodes with high centrality to form tightly interconnected communities (for reviews see: [2], [9], [68]). Furthermore, network analysis in neurophysiological studies is often performed in different frequency bands. In our study, we chose to include only theta frequency derived networks. Most studies have repeatedly identified epilepsy specific changes in this frequency band [11]. Other frequency bands, however, might contain additional information on epilepsy related network changes that were not considered in our study. Finally, the included studies were diverse in modality, network size and analysis strategy. This heterogeneity relates to the inconsistency between individual study results. Some studies combined different modalities into one study and revealed inconsistent results [30], [69]. For that reason, we performed subanalysis for functional and structural studies. We found a less integrated network organization in patients, both in functional and structural networks, as compared to controls. A more segregated network in patients was found for structural networks only. This might indicate that different mechanisms (or detection sensitivities) are at play for structural and functional network alterations in patients with epilepsy. No differences were found between the epilepsy patients’ functional network data and patients’ structural network data (Figs. 5 and 6). It is therefore not possible to make an unambiguous statement on the differences between functional and structural networks and if these networks interact. Studies investigating functional and structural networks concomitantly are needed to clarify the similarities or discrepancy between modalities [30], [69].

A difference in the number of network nodes will influence network metrics [20]. Also, different analysis strategies might affect the eventual outcome of network metrics [11], [70]. These differences complicate direct comparison of individual study results. However, using the standardized mean difference in our meta-analysis, we intended to correct for scale differences between studies due to modality, connectivity measures and network construction or size. Another important issue, related to our hypothesis, is our implicit assumption that network alterations in focal epilepsies are similar, regardless their etiology. One could argue that this is not the case, as studies have revealed differences in network connectivity and topology for patients with different etiologies [25], [26]. Unfortunately, we were not able to investigate etiology-related differences in network topology due to missing information in most of studies.

In conclusion, this systematic review and meta-analysis revealed that interictal whole brain networks are characterized by a less integrated and more segregated organization in focal epilepsies. A large, sufficiently powered study is required to confirm our findings. Future work may focus on *i*) pinpointing the exact cause of changes in brain-wide network organization, *ii*) identification of relevant effect modifiers such as antiepileptic therapy, duration of epilepsy or type of epilepsy and *iii*) characterizing network differences in specific subgroups of focal epilepsy using a range of features beyond measures that quantify network integration and segregation. When interested in functional connectivity, we encourage using more than one connectivity measure to investigate the robustness of the possible network alterations found in patients with epilepsy. Finally, when attempting to unravel causality in the relation between epileptic seizures and network alterations in patients with epilepsy, we suggest the use of longitudinal studies and a systematic evaluation and interpretation of the data according to guidelines how to draw a conclusion on causality [71].

## Supporting Information

S1 Data **RevMan files.**
(RM5)

S1 File Containing the following tables and figures: S1 Table. Search strategy. S2 Table. Additional methodological information on the EEG and functional MRI studies. S3 Table. Study quality assessment. S4 Table. Sensitivity analysis. S1 Figure. Funnel plot of average path length for individual studies. S2 Figure. Funnel plot of average clustering coefficient for individual studies. S3a Figure. Meta-analysis of the average path length according to age. S3b Figure. Meta-analysis of the average clustering coefficient according to age. S4a Figure. Meta-analysis of the average path length according to type of epilepsy. S4b Figure. Meta-analysis of the average clustering coefficient according to type of epilepsy.
(PDF)
