# Supplementary material for: Brain Network Organization in Focal Epilepsy: A Systematic Review and Meta-Analysis
Source: PLoS One. 2014 Dec 10;9(12):e114606. doi: 10.1371/journal.pone.0114606 (PMC4262431; doi:10.1371/journal.pone.0114606)
Supplement: S1 File — Containing the following tables and figures: S1 Table. Search strategy. S2 Table. Additional methodological information on the EEG and functional MRI studies. S3 Table. Study quality assessment. S4 Table. Sensitivity analysis. S1 Figure. Funnel plot of average path length for individual studies. S2 Figure. Funnel plot of average clustering coefficient for individual studies. S3a Figure. Meta-analysis of the average path length according to age. S3b Figure. Meta-analysis of the average clustering coefficient according to age. S4a Figure. Meta-analysis of the average path length according to type of epilepsy. S4b Figure. Meta-analysis of the average clustering coefficient according to type of epilepsy. (PDF) [file pone.0114606.s002.pdf]

Supporting Information

Pubmed (NCBI) Search
((TS=(epilepsy OR seizure OR convulsion) AND TS=(DTI OR "diffusion tensor imaging" OR "diffusion magnetic resonance imaging" OR "diffusion weighted imaging" OR EEG OR electroencephalogram OR MEG OR magnetoencephalogram OR fMRI OR "functional MRI" OR "functional magnetic resonance imaging" OR sMRI OR "structural MRI" OR "structural magnetic resonance imaging" OR "cortical thickness") AND TS=(Network OR "network analysis" OR "network theoretical analysis" OR "network-theoretical analysis" OR "graph analysis" OR "graph theoretical analysis" OR "graph-theoretical analysis" OR "clustering coefficient" OR "path length" OR "shortest path" OR "small-world index" OR "small world index" OR "small-worldness" OR "small worldness" OR "local efficiency" OR "global efficiency" OR segregation OR integration OR topology))) AND Language=(English)<br><br>*Databases=SCI-EXPANDED, CPCI-S Timespan=All Years*
**ISI Web of Science (Thomson Reuters) search**
((TS=(epilepsy OR seizure OR convulsion) AND TS=(DTI OR "diffusion tensor imaging" OR "diffusion magnetic resonance imaging" OR "diffusion weighted imaging" OR EEG OR electroencephalogram OR MEG OR magnetoencephalogram OR fMRI OR "functional MRI" OR "functional magnetic resonance imaging" OR sMRI OR "structural MRI" OR "structural magnetic resonance imaging" OR "cortical thickness") AND TS=(Network OR "network analysis" OR "network theoretical analysis" OR "network-theoretical analysis" OR "graph analysis" OR "graph theoretical analysis" OR "graph-theoretical analysis" OR "clustering coefficient" OR "path length" OR "shortest path" OR "small-world index" OR "small world index" OR "small-worldness" OR "small worldness" OR "local efficiency" OR "global efficiency" OR segregation OR integration OR topology))) AND Language=(English)<br>Refined by: Web of Science Categories=(CLINICAL NEUROLOGY OR NEUROIMAGING)<br><br>*Timespan=All Years. Databases=SCI-EXPANDED, CPCI-S*
**Embase (Excerpta Medica Database) search**
((TS=(epilepsy OR seizure OR convulsion) AND TS=(DTI OR "diffusion tensor imaging" OR "diffusion magnetic resonance imaging" OR "diffusion weighted imaging" OR EEG OR electroencephalogram OR MEG OR magnetoencephalogram OR fMRI OR "functional MRI" OR "functional magnetic resonance imaging" OR sMRI OR "structural MRI" OR "structural magnetic resonance imaging" OR "cortical thickness") AND TS=(Network OR "network analysis" OR "network theoretical analysis" OR "network-theoretical analysis" OR "graph analysis" OR "graph theoretical analysis" OR "graph-theoretical analysis" OR "clustering coefficient" OR "path length" OR "shortest path" OR "small-world index" OR "small world index" OR "small-worldness" OR "small worldness" OR "local efficiency" OR "global efficiency" OR segregation OR integration OR topology))) AND Language=(English)<br>Refined by: Research Areas=(NEUROSCIENCES NEUROLOGY)<br><br>*Databases=SCI-EXPANDED, CPCI-S Timespan=All Years*

Table S1. Search strategy

Search strategies for the online databases Pubmed (NCBI), ISI Web of Science (Thomson Reuters) and Embase (Excerpta Medica Database).

Study	Modality	Recording characteristics	Condition	Reference	Artifact management
Bartolomei et al., 2006	MEG	SF: 312.5 Hz<br>Bandpass filter of 0.25-125 Hz	Awake, no-task, EC	n.a.	Selection of artifact free epochs by visual analysis
Bosma et al., 2009	MEG	SF: 312.5 Hz<br>Bandpass filter of 0.25-125 Hz	Awake, no-task, EC	n.a.	Selection of artifact free epochs by visual analysis
Horstmann et al., 2010	EEG	SF: 254.31 Hz<br>Bandpass filter of 0-50 Hz	Awake, no-task,15 min EO, 15 min EC Acoustical instruction to open/close eyes	Right mastoid and average common reference	EOG to record ocular artifacts<br>Wavelet-based correction scheme to minimize influence of technical (e.g., amplifier resets) and physiologic artifacts (e.g., eye movements/blinks or head movements)
Liao et al., 2010	fMRI	FS: 1.5 T<br>Echoplanar imaging sequence	Awake, no-task, EC	T1-weighted image for anatomic reference	First 10 images discarded to ensure magnetization equilibrium<br>Other images: acquisition time-delay and head motion correction, translation or rotation > +1 mm or +10 = excluded
Quraan et al., 2013	EEG	SF: 500 Hz<br>Bandpass filter of 2-20 Hz	Awake, no-task, 3 min EO, 3 min EC	Average common reference	EOG to record ocular artifacts<br>Manual removal remaining artifacts
Vaessen et al., 2013	fMRI	FS: 3.0 T<br>Echoplanar imaging sequence	n.r.	T1-weigthed 3D fast field echo for anatomic reference	Correction for motion artifacts and high frequency noise
Van Dellen et al., 2012	MEG	SF: 625 Hz<br>Bandpass filter of 0.25-125 Hz	Awake, no-task, EC	n.a.	Selection of artifact free epochs by visual analysis
Van Diessen et al., 2013	EEG	SF: 512 Hz<br>Bandpass filter of 0.16-70 Hz	Awake, no-task, EC	Average reference	Removal of Fp1, Fp2, A1, and A2 electrodes to minimize eye- movement artifacts<br>Selection of artifact free epochs by visual analysis
Vlooswijk et al., 2011	fMRI	FS: 3.0 T<br>Echoplanar imaging sequence	Awake, 6 times 30s word-paradigm, 6 times 30s no-task resting state	T1-weigthed 3D fast field echo for anatomic reference	Acquisition time-delay and head motion correction

Table S2. Additional methodological information on the encephalographic and functional MRI studies

SF = sample frequency, FS = field strength, Hz = hertz, T = tesla, EC = eyes closed, EO = eyes open, EOG = electrooculography, n.a. = not applicable, n.r. = not reported, Fp = frontopolar, A = auricular.

Study	Quality indicators from the Newcastle-Ottawa Scale [67]							
	Selection				Comparability		Exposure	Total score
	1	2	3	4	5A	5B	6	
Bartolomei et al., 2006	*		*	*		*	*	5
Bernhardt et al., 2011	*	*	*	*	*	*	*	7
Bonilha et al., 2012	*	*	*	*	*	*	*	7
Bosma et al., 2009	*	*	*	*	*	*	*	7
Horstmann et al., 2010	*	*	*	*		*	*	6
Liao et al., 2010	*	*	*	*		*	*	6
Quraan et al., 2013	*	*	*	*			*	5
Raj et al., 2010	*	*	*		*	*	*	6
Vaessen et al., 2012	*	*	*	*		*	*	6
Vaessen et al., 2013	*	*	*	*	*		*	6
van Dellen et al., 2012	*	*	*	*		*	*	6
van Diessen et al., 2013	*	*	*	*	*	*	*	7
Vlooswijk et al., 2011	*	*	*	*			*	5

Table S3. Study quality assessment.

The table presents the Newcastle-Ottawa Scale quality assessment categories. These categories are included: 1 = cases independently validated; 2 = cases are representative of the population; 3 = community controls; 4 = controls have no history of focal epilepsy; 5A = study controls for age; 5B = study controls for additional factors; 6 = ascertainment of exposure by blinded interview or secure record.

	Average path length				Average clustering coefficient			
	I^2 (%)	p-value	SMD (CI)	p-value	I^2 (%)	p-value	SMD (CI)	p-value
None (= weighted; high sparsity level)	0	0.57	0.29 (0.12, 0.45)	0.0007	73	<0.0001	0.35 (0.05, 0.65)	0.02
Network direction:								
Binary instead of weighted[i]	8	0.37	0.22 (0.05, 0.38)	0.009	75	<0.00001	0.32 (0.00, 0.63)	0.05
Sparsity level[ii]:								
Low	0	0.60	0.24 (0.08, 0.40)	0.003	72	<0.0001	0.36 (0.06, 0.65)	0.02
Middle	0	0.65	0.23 (0.08, 0.39)	0.003	73	<0.0001	0.34 (0.03, 0.64)	0.03

Table S4. Sensitivity analysis

The robustness of the summary estimates is presented as recalculations of the summary estimates at different network sparsity levels and for binary or weighted networks. [i] In the analysis of network directionality, we used the binary instead of weighted data from the average clustering coefficient and average path length provided by [30] and [42]; [ii] In the sparsity level analyses we used data from low and middle instead of high sparsity levels provided by [21], [39], and [42]. SMD = standardized mean difference, CI = 95 % confidence interval.

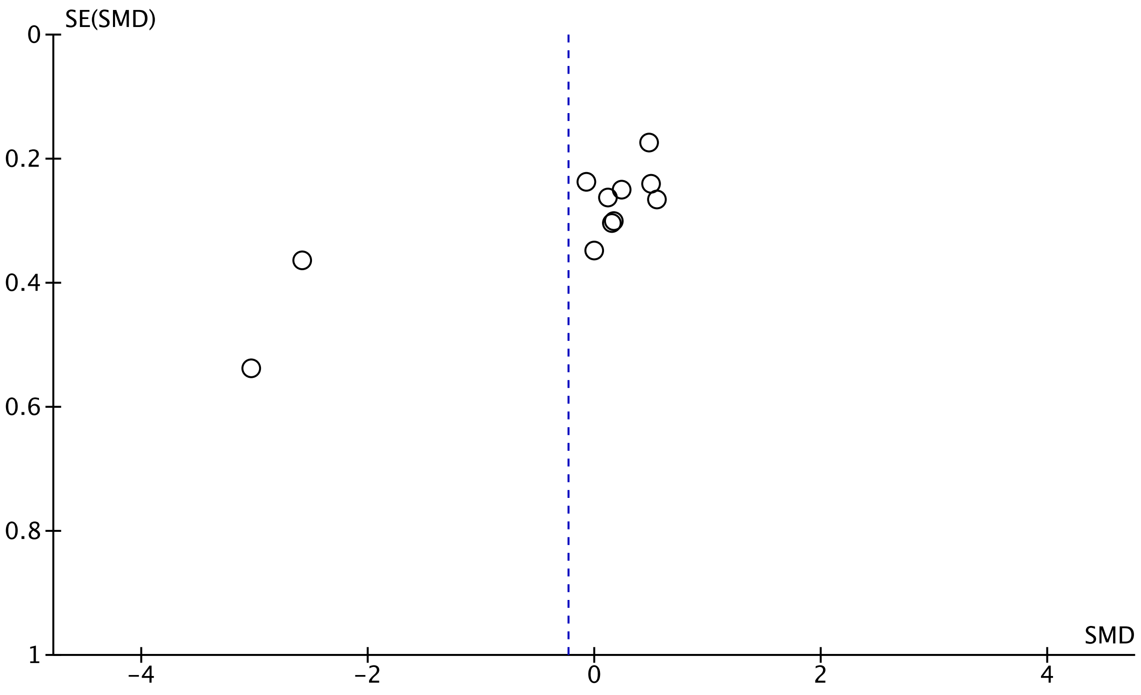

Figure S1. Funnel plot of average path length for individual studies

A funnel plot for the standardized mean difference (SMD) average path length estimates from individual studies against the standard error (SE) of the SMD as a measure of the study's precision. The dashed vertical line corresponds to the overall summary SMD estimate.

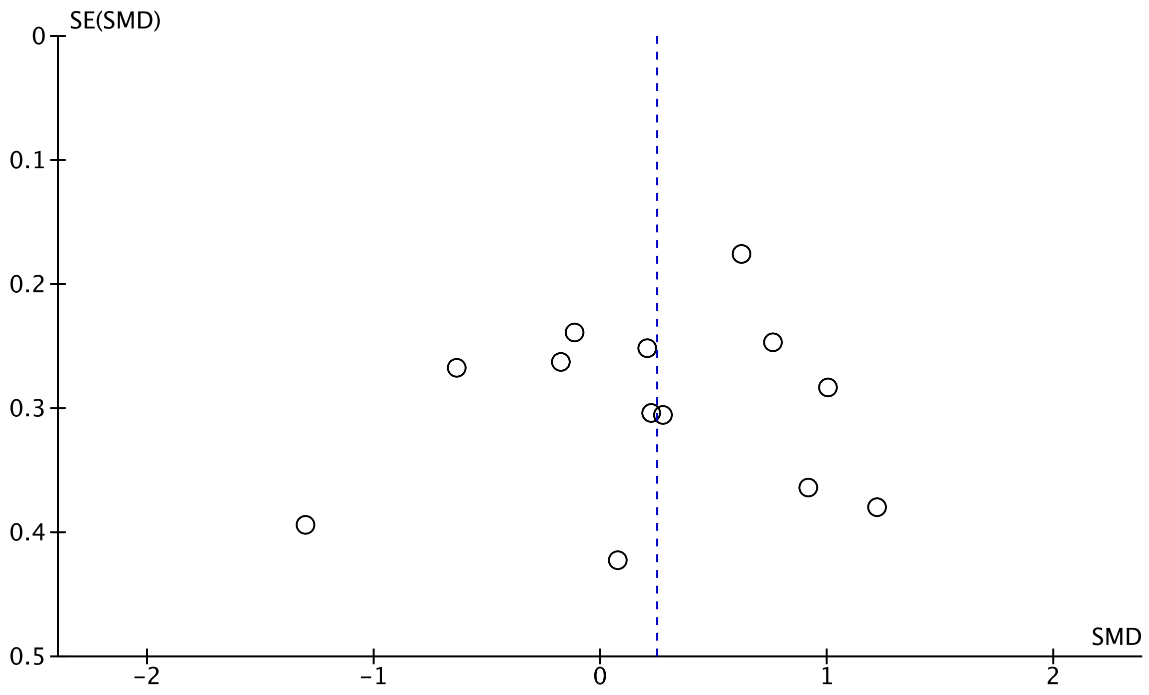

Figure S2. Funnel plot of average clustering coefficient for individual studies

A funnel plot for the standardized mean difference (SMD) average clustering coefficient estimates from individual studies against the standard error (SE) of the SMD as a measure of the study's precision. The dashed vertical line corresponds to the overall summary SMD estimate.

Study or Subgroup	Focal epilepsy patients Mean	SD	Total	Control Mean	SD	Total	Weight	Std. Mean Difference IV, Random, 95% CI
**Adults**								
Bernhardt et al. 2011	2	0.104	122	1.94	0.16	47	23.3%	0.49 [0.15, 0.83]
Bonilha et al. 2012	1.34	0.03	12	1.34	0.03	26	5.8%	0.00 [−0.68, 0.68]
Horstmann et al. 2010	1.06	0.028	21	1.055	0.029	23	7.7%	0.17 [−0.42, 0.76]
Liao et al. 2010	2.095	0.36	18	2.037	0.38	27	7.6%	0.15 [−0.44, 0.75]
Vaessen et al. 2012	62.5	14.7	39	60.8	10.8	23	10.2%	0.13 [−0.39, 0.64]
Van Dellen et al. 2012	1.097	0.022	35	1.087	0.018	36	12.1%	0.49 [0.02, 0.97]
Vlooswijk et al. 2011	1.44	0.083	41	1.39	0.101	23	10.0%	0.55 [0.03, 1.07]
**Subtotal (95% CI)**			**288**			**205**	**76.6%**	**0.35 [0.16, 0.54]**
Heterogeneity: $Tau^2 = 0.00$; $Chi^2 = 4.06$, df = 6 (P = 0.67); $I^2 = 0\%$								
Test for overall effect: Z = 3.63 (P = 0.0003)								
**Children**								
Vaessen et al. 2013	0.52	0.074	28	0.504	0.055	37	11.1%	0.25 [−0.25, 0.74]
Van Diessen et al. 2013	1.051	0.075	35	1.055	0.04	35	12.3%	−0.07 [−0.53, 0.40]
**Subtotal (95% CI)**			**63**			**72**	**23.4%**	**0.08 [−0.26, 0.42]**
Heterogeneity: $Tau^2 = 0.00$; $Chi^2 = 0.82$, df = 1 (P = 0.37); $I^2 = 0\%$								
Test for overall effect: Z = 0.48 (P = 0.63)								
**Total (95% CI)**			**351**			**277**	**100.0%**	**0.29 [0.12, 0.45]**
Heterogeneity: $Tau^2 = 0.00$; $Chi^2 = 6.66$, df = 8 (P = 0.57); $I^2 = 0\%$								
Test for overall effect: Z = 3.41 (P = 0.0007)								
Test for subgroup differences: $Chi^2 = 1.78$, df = 1 (P = 0.18), $I^2 = 44.0\%$								

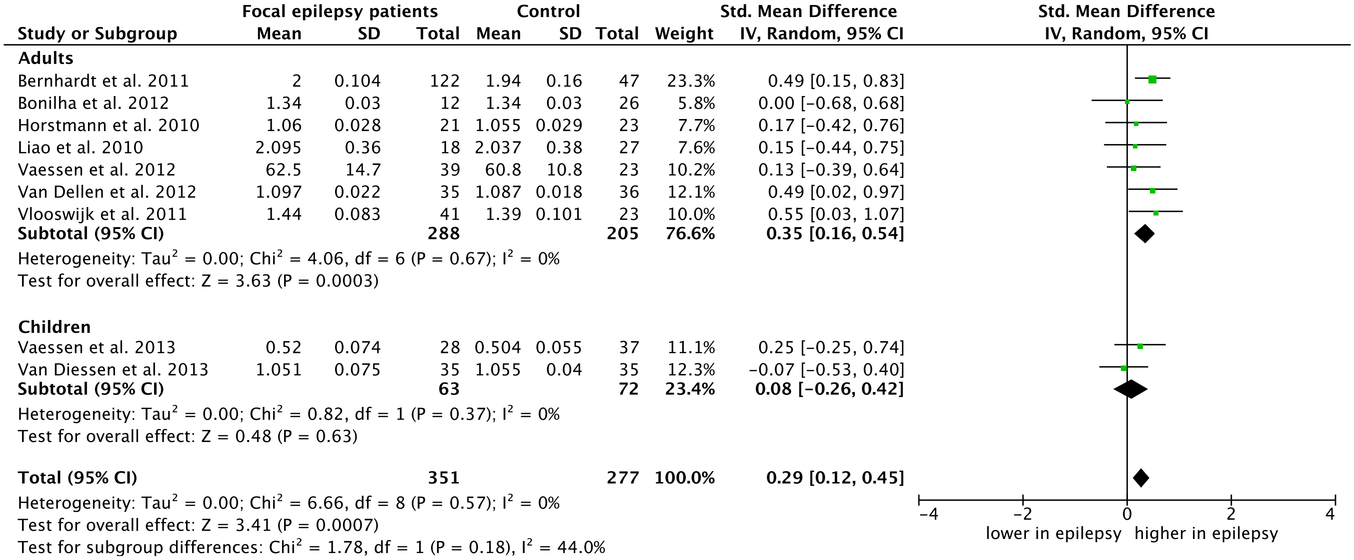

Figure S3a. Meta-analysis of the average path length according to age

The forest plot displays the standardized mean differences (SMD) between focal epilepsy patients and controls with the 95% confidence intervals (CI) for the network studies reporting the average path length. Studies are divided in two categories: studies on children and studies on adults. No difference between patients and controls is specified with a vertical line at 0. The overall pooled SMD for the average path length of adults was 0.35 (CI: 0.16 to 0.54, p = 0.0003) and 0.08 (CI: -0.26 to 0.42, p = 0.63) for the path length of children. The SMDs of these subgroups were not statistically different (p = 0.18, $I^2 = 44.0\%$).

Study or Subgroup	Focal epilepsy patients Mean	SD	Total	Controls Mean	SD	Total	Weight	Std. Mean Difference IV, Random, 95% CI
**Adults**								
Bernhardt et al. 2011	0.4	0.108	122	0.31	0.21	47	10.3%	0.62 [0.28, 0.97]
Bonilha et al. 2012	0.72	0.016	12	0.7	0.016	26	6.9%	1.22 [0.48, 1.97]
Bosma et al. 2009	2.42	0.64	17	1.886	0.482	17	7.1%	0.92 [0.21, 1.63]
Horstmann et al. 2010	0.693	0.023	21	0.679	0.08	23	8.1%	0.23 [−0.36, 0.82]
Liao et al. 2010	2.221	0.97	18	1.971	0.82	27	8.1%	0.28 [−0.32, 0.88]
Quraan et al. 2013	1.233	0.333	9	1.217	0.017	15	6.3%	0.08 [−0.75, 0.90]
Raj et al. 2010	0.486	0.076	27	0.408	0.077	30	8.5%	1.01 [0.45, 1.56]
Vaessen et al. 2012	1.75	0.35	39	1.81	0.34	23	8.8%	−0.17 [−0.69, 0.35]
Van Dellen et al. 2012	1.066	0.014	35	1.056	0.012	36	9.1%	0.76 [0.28, 1.24]
Vlooswijk et al. 2011	4.48	2.004	41	5.69	1.655	23	8.7%	−0.63 [−1.16, −0.11]
**Subtotal (95% CI)**			**341**			**267**	**81.8%**	**0.42 [0.07, 0.77]**
Heterogeneity: $Tau^2 = 0.23$; $Chi^2 = 35.83$, df = 9 ($P < 0.0001$); $I^2 = 75\%$								
Test for overall effect: Z = 2.34 (P = 0.02)								
**Children**								
Vaessen et al. 2013	2.26	0.339	28	2.2	0.243	37	9.0%	0.21 [−0.29, 0.70]
Van Diessen et al. 2013	1.006	0.01	35	1.007	0.007	35	9.2%	−0.11 [−0.58, 0.35]
**Subtotal (95% CI)**			**63**			**72**	**18.2%**	**0.04 [−0.30, 0.38]**
Heterogeneity: $Tau^2 = 0.00$; $Chi^2 = 0.85$, df = 1 ($P = 0.36$); $I^2 = 0\%$								
Test for overall effect: Z = 0.22 (P = 0.83)								
**Total (95% CI)**			**404**			**339**	**100.0%**	**0.35 [0.05, 0.65]**
Heterogeneity: $Tau^2 = 0.20$; $Chi^2 = 40.59$, df = 11 ($P < 0.0001$); $I^2 = 73\%$								
Test for overall effect: Z = 2.29 (P = 0.02)								
Test for subgroup differences: $Chi^2 = 2.35$, df = 1 ($P = 0.13$), $I^2 = 57.5\%$								

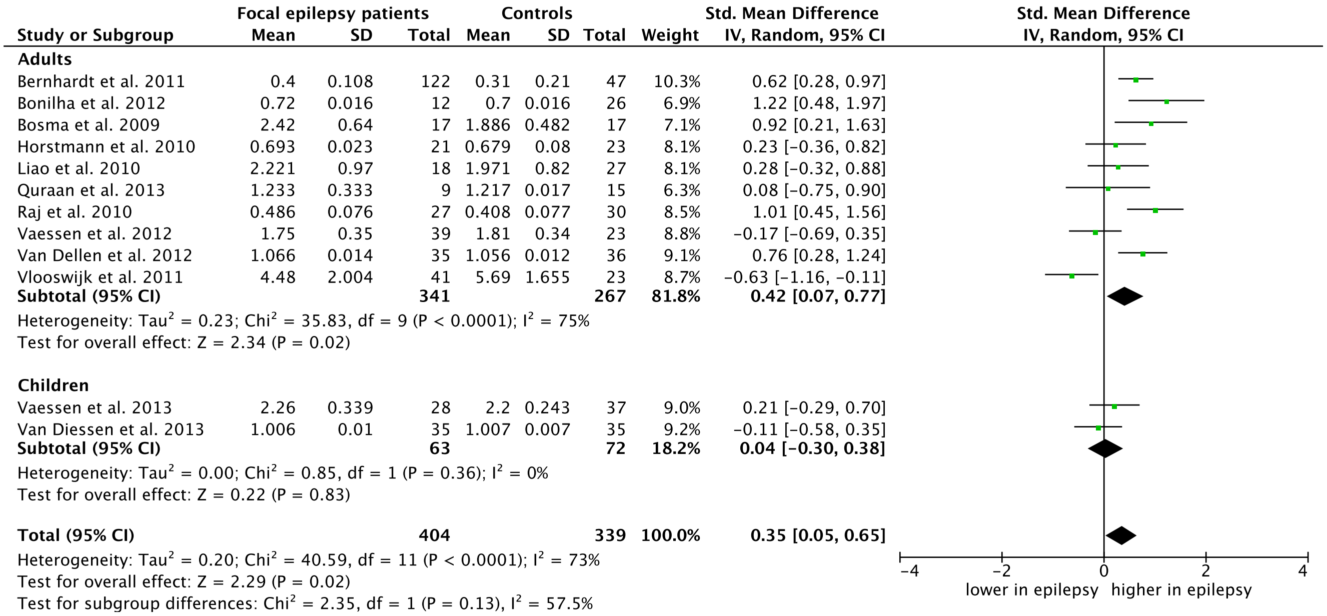

Figure S3b. Meta-analysis of the average clustering coefficient according to age

The forest plot displays the standardized mean differences (SMD) between focal epilepsy patients and controls with the 95% confidence intervals (CI) for network studies reporting the average clustering coefficient. Studies are divided in two categories: studies on children and studies on adults. No difference between patients and controls is specified with a vertical line at 0. The overall pooled SMD for the clustering coefficient of adults was 0.42 (CI: 0.07 to 0.77, $p < 0.0001$) and 0.04 (CI: -0.30 to 0.38, $p = 0.83$) for the average path length of children. The SMDs of these subgroups were not statistically different ($p = 0.13$, $I^2 = 57.5\%$).

Study or Subgroup	Focal epilepsy patients Mean	SD	Total	Control Mean	SD	Total	Weight	Std. Mean Difference IV, Random, 95% CI
**Temporal**								
Bernhardt et al. 2011	2	0.104	122	1.94	0.16	47	23.3%	0.49 [0.15, 0.83]
Bonilha et al. 2012	1.34	0.03	12	1.34	0.03	26	5.8%	0.00 [−0.68, 0.68]
Liao et al. 2010	2.095	0.36	18	2.037	0.38	27	7.6%	0.15 [−0.44, 0.75]
**Subtotal (95% CI)**			**152**			**100**	**36.6%**	**0.34 [0.06, 0.62]**
Heterogeneity: Tau^2 = 0.00; Chi^2 = 2.07, df = 2 (P = 0.36); I^2 = 3%								
Test for overall effect: Z = 2.37 (P = 0.02)								
**Extratemporal**								
Horstmann et al. 2010	1.06	0.028	21	1.055	0.029	23	7.7%	0.17 [−0.42, 0.76]
Vaessen et al. 2012	62.5	14.7	39	60.8	10.8	23	10.2%	0.13 [−0.39, 0.64]
Vaessen et al. 2013	0.52	0.074	28	0.504	0.055	37	11.1%	0.25 [−0.25, 0.74]
Van Diessen et al. 2013	1.051	0.075	35	1.055	0.04	35	12.3%	−0.07 [−0.53, 0.40]
Vlooswijk et al. 2011	1.44	0.083	41	1.39	0.101	23	10.0%	0.55 [0.03, 1.07]
**Subtotal (95% CI)**			**164**			**141**	**51.3%**	**0.20 [−0.03, 0.43]**
Heterogeneity: Tau^2 = 0.00; Chi^2 = 3.10, df = 4 (P = 0.54); I^2 = 0%								
Test for overall effect: Z = 1.67 (P = 0.09)								
**Tumor (secondary epilepsy)**								
Van Dellen et al. 2012	1.097	0.022	35	1.087	0.018	36	12.1%	0.49 [0.02, 0.97]
**Subtotal (95% CI)**			**35**			**36**	**12.1%**	**0.49 [0.02, 0.97]**
Heterogeneity: Not applicable								
Test for overall effect: Z = 2.04 (P = 0.04)								
**Total (95% CI)**			**351**			**277**	**100.0%**	**0.29 [0.12, 0.45]**
Heterogeneity: Tau^2 = 0.00; Chi^2 = 6.66, df = 8 (P = 0.57); I^2 = 0%								
Test for overall effect: Z = 3.41 (P = 0.0007)								
Test for subgroup differences: Chi^2 = 1.46, df = 2 (P = 0.48), I^2 = 0%								

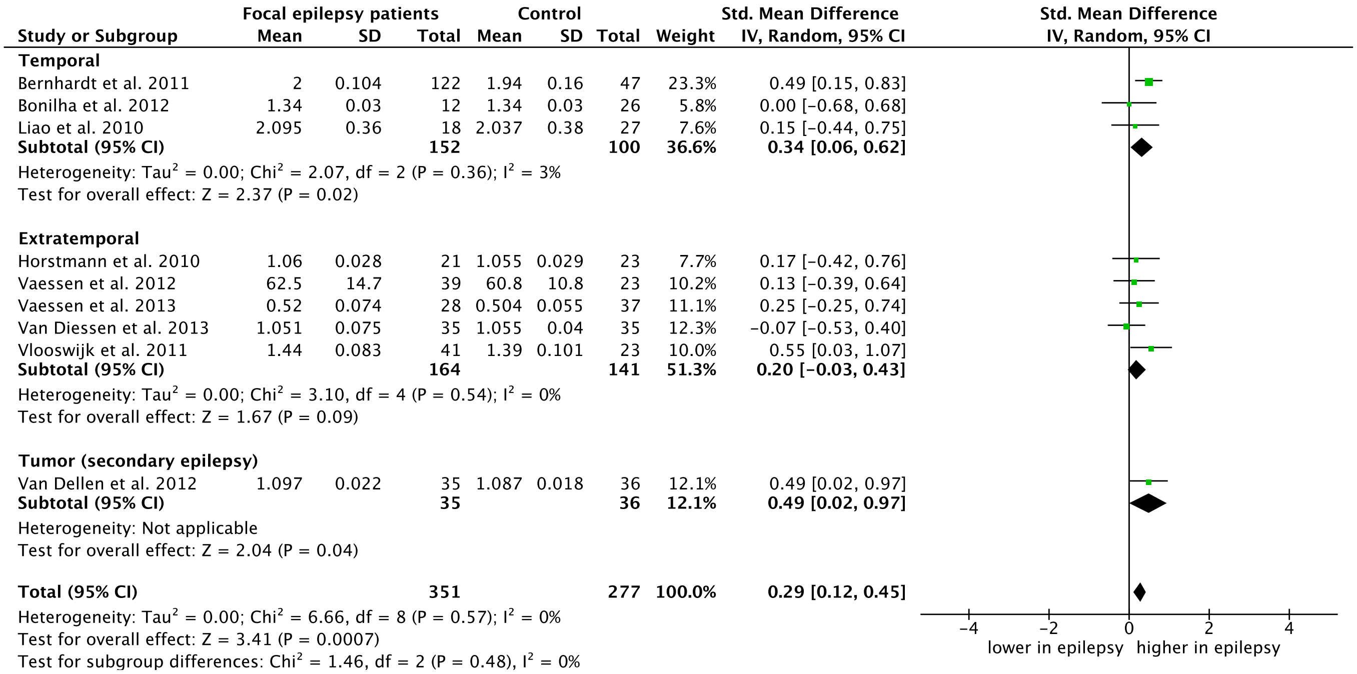

Figure S4a. Meta-analysis of the average path length according to type of epilepsy

The forest plot displays the standardized mean differences (SMD) between focal epilepsy patients and controls with the 95% confidence intervals (CI) for the network studies reporting the average path length. Results are separated for studies that included only temporal lobe epilepsy patients, extratemporal or tumor related epilepsy. No difference between patients and controls is specified with a vertical line at 0. The overall pooled SMD for the average path length of studies that investigated temporal lobe epilepsy was 0.34 (CI: 0.06 to 0.62, p = 0.02), 0.20 (CI: -0.03 to 0.43, p = 0.09) for extratemporal epilepsy. Only one study was included for tumor related epilepsy. The SMDs of these subgroups were not statistically different (p = 0.48, I^2 = 0%).

Study or Subgroup	Focal epilepsy patients Mean	SD	Total	Controls Mean	SD	Total	Weight	Std. Mean Difference IV, Random, 95% CI
**Temporal**								
Bernhardt et al. 2011	0.4	0.108	122	0.31	0.21	47	10.3%	0.62 [0.28, 0.97]
Bonilha et al. 2012	0.72	0.016	12	0.7	0.016	26	6.9%	1.22 [0.48, 1.97]
Liao et al. 2010	2.221	0.97	18	1.971	0.82	27	8.1%	0.28 [−0.32, 0.88]
Quraan et al. 2013	1.233	0.333	9	1.217	0.017	15	6.3%	0.08 [−0.75, 0.90]
Raj et al. 2010	0.486	0.076	27	0.408	0.077	30	8.5%	1.01 [0.45, 1.56]
**Subtotal (95% CI)**			**188**			**145**	**39.9%**	**0.66 [0.31, 1.00]**
Heterogeneity: $Tau^2 = 0.07$; $Chi^2 = 7.21$, df = 4 (P = 0.13); $I^2 = 45\%$								
Test for overall effect: Z = 3.72 (P = 0.0002)								
**Extratemporal**								
Horstmann et al. 2010	0.693	0.023	21	0.679	0.08	23	8.1%	0.23 [−0.36, 0.82]
Vaessen et al. 2012	1.75	0.35	39	1.81	0.34	23	8.8%	−0.17 [−0.69, 0.35]
Vaessen et al. 2013	2.26	0.339	28	2.2	0.243	37	9.0%	0.21 [−0.29, 0.70]
Van Diessen et al. 2013	1.006	0.01	35	1.007	0.007	35	9.2%	−0.11 [−0.58, 0.35]
Vlooswijk et al. 2011	4.48	2.004	41	5.69	1.655	23	8.7%	−0.63 [−1.16, −0.11]
**Subtotal (95% CI)**			**164**			**141**	**43.8%**	**−0.10 [−0.40, 0.20]**
Heterogeneity: $Tau^2 = 0.05$; $Chi^2 = 6.73$, df = 4 (P = 0.15); $I^2 = 41\%$								
Test for overall effect: Z = 0.67 (P = 0.50)								
**Tumor (secondary epilepsy)**								
Bosma et al. 2009	2.42	0.64	17	1.886	0.482	17	7.1%	0.92 [0.21, 1.63]
Van Dellen et al. 2012	1.066	0.014	35	1.056	0.012	36	9.1%	0.76 [0.28, 1.24]
**Subtotal (95% CI)**			**52**			**53**	**16.2%**	**0.81 [0.41, 1.21]**
Heterogeneity: $Tau^2 = 0.00$; $Chi^2 = 0.13$, df = 1 (P = 0.71); $I^2 = 0\%$								
Test for overall effect: Z = 3.98 (P < 0.0001)								
**Total (95% CI)**			**404**			**339**	**100.0%**	**0.35 [0.05, 0.65]**
Heterogeneity: $Tau^2 = 0.20$; $Chi^2 = 40.59$, df = 11 (P < 0.0001); $I^2 = 73\%$								
Test for overall effect: Z = 2.29 (P = 0.02)								
Test for subgroup differences: $Chi^2 = 16.87$, df = 2 (P = 0.0002), $I^2 = 88.1\%$								

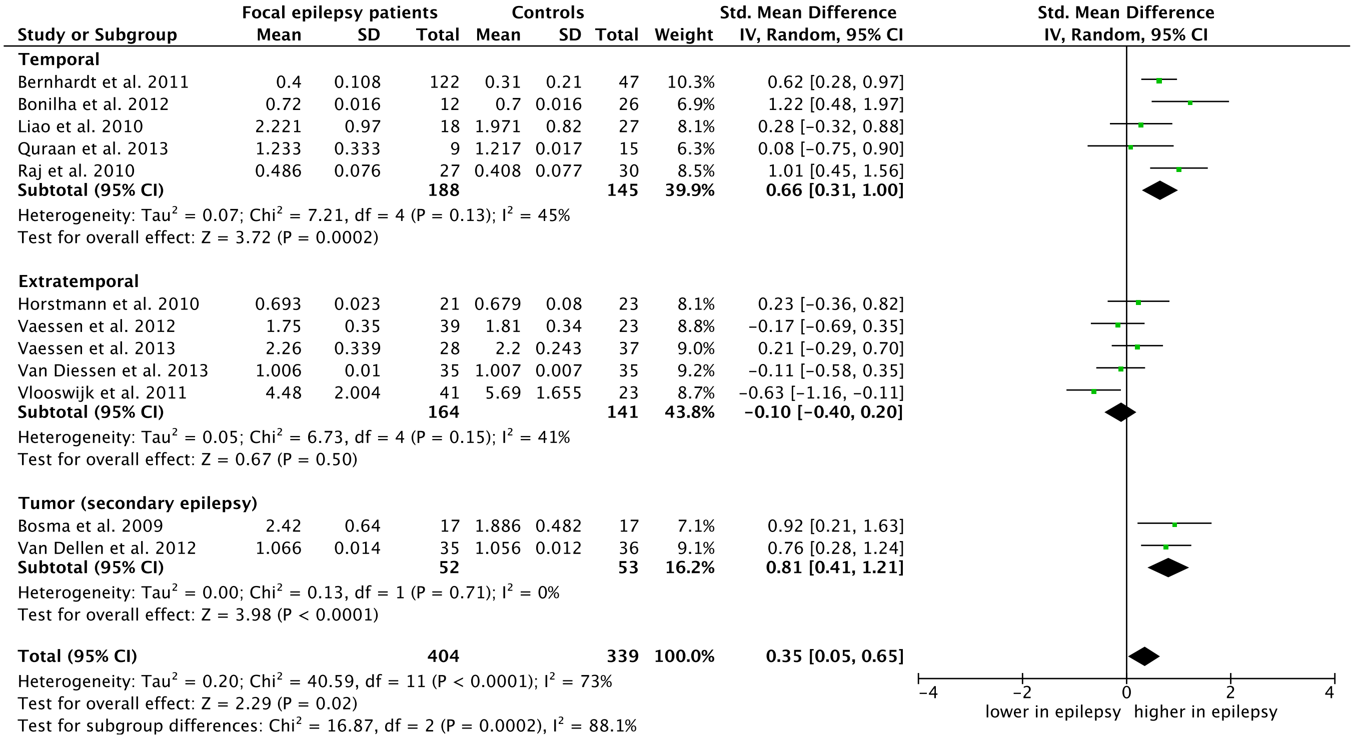

Figure S4b. Meta-analysis of the average clustering coefficient according to type of epilepsy

The forest plot displays the standardized mean differences (SMD) between focal epilepsy patients and controls with the 95% confidence intervals (CI) for the network studies reporting the average clustering coefficient. Results are separated for studies that included only temporal lobe epilepsy patients, extratemporal or tumor related epilepsy. No difference between patients and controls is specified with a vertical line at 0. The overall pooled SMD for the average clustering coefficient of studies that investigated temporal lobe epilepsy was 0.66 (CI: 0.31 to 1.00, p = 0.0002), -0.10 (CI: -0.40 to 0.20, p = 0.50) for extratemporal epilepsy and 0.81 (CI: 0.41 to 1.21, p < 0.0001) for tumor related epilepsy. The SMDs of these subgroups were statistically different (p = 0.0002), but due to a considerable heterogeneity (I^2 =88.1%), not reliable.
